# Extranodal Marginal Zone Mucosa-Associated Lymphoid Tissue Lymphoma of the Gallbladder: A Case Report and Literature Review

**DOI:** 10.7759/cureus.35825

**Published:** 2023-03-06

**Authors:** Preeti Farmah, Apoorva H Mehta, Alexander H Vu, Loic S Tchokouani, Sharique Nazir

**Affiliations:** 1 Department of Minimally Invasive Surgery/General Surgery, NYU Langone Hospital - Brooklyn, Brooklyn, USA; 2 Department of General Surgery, Harlem Hospital Center, Columbia University College of Physicians and Surgeons, New York, USA

**Keywords:** cholelithiasis, chronic cholecystitis, non-hodgkin’s lymphoma, gallbladder lymphoma, gallbladder malt lymphoma, extranodal marginal zone malt lymphoma

## Abstract

We describe the case of a patient with extranodal marginal zone mucosa-associated lymphoid tissue (MALT) lymphoma of the gallbladder discovered incidentally after elective cholecystectomy. A 76-year-old female with a history of non-Hodgkin’s lymphoma of the right breast and rectal cancer stage Tis requiring trans-anal excision presented with chronic intermittent abdominal pain. Computed tomography (CT) scan showed multiple calcified gallstones impacted in the gallbladder, with no evidence of enlarging lymphadenopathy indicating an elective cholecystectomy. The intra- and post-operative courses were unremarkable, but pathology review revealed immunohistochemistry positive for CD20 and BCL-2 with a Ki67 proliferation index of 5%, which was diagnostic of extranodal marginal zone MALT lymphoma of the gallbladder. The patient was followed up by a medical oncologist, and after extensive discussion, the decision was made to continue observation with close monitoring without systemic chemotherapy given the asymptomatic presentation. We also examined the pertinent literature to MALT lymphoma of the gallbladder and discussed theories suggested for its pathophysiology.

## Introduction

Gallbladder and bile duct lymphomas are rare occurrences, with only around 52 cases reported in the literature [1]. Extranodal marginal zone mucosa-associated lymphoid tissue (MALT) lymphoma is especially rare, with approximately six reported cases [1-2]. The most common presentation mimics acute cholecystitis with abdominal pain, fever, nausea, and vomiting. Given the gallbladder's lack of lymphoid tissue, multiple theories attempt to explain the pathophysiology of disease progression, ranging from lymphocytic proliferation secondary to chronic inflammation to antigenic stimulation inducing chromosomal translocation.

We describe the case of a patient with extranodal marginal zone MALT lymphoma of the gallbladder incidentally revealed on histopathology after an elective cholecystectomy. The patient was a 76-year-old female who presented with chronic intermittent abdominal pain and was found to have multiple gallstones on imaging. This patient has previously diagnosed non-Hodgkin’s lymphoma of the right breast and rectal cancer stage Tis requiring trans-anal excision.

To our knowledge, this is one of the few case reports describing extranodal marginal zone MALT lymphoma of the gallbladder with a unique patient medical history.

## Case presentation

A 76-year-old female with a history of right breast non-Hodgkin’s lymphoma and rectal cancer stage Tis requiring trans-anal excision presented for elective cholecystectomy after CT imaging revealed multiple gallstones.

Regarding her history of breast lymphoma, the patient was found to have an abnormal 7-mm mass found on screening mammography. Fine needle aspiration (FNA) with flow cytometry revealed a phenotype of non-Hodgkin’s lymphoma. A positron-emission tomography (PET) scan showed non-specific, generalized lymphadenopathy. She was asymptomatic and therefore managed by her medical oncologist with observation and serial imaging without systemic chemotherapy.

**Figure 1:**
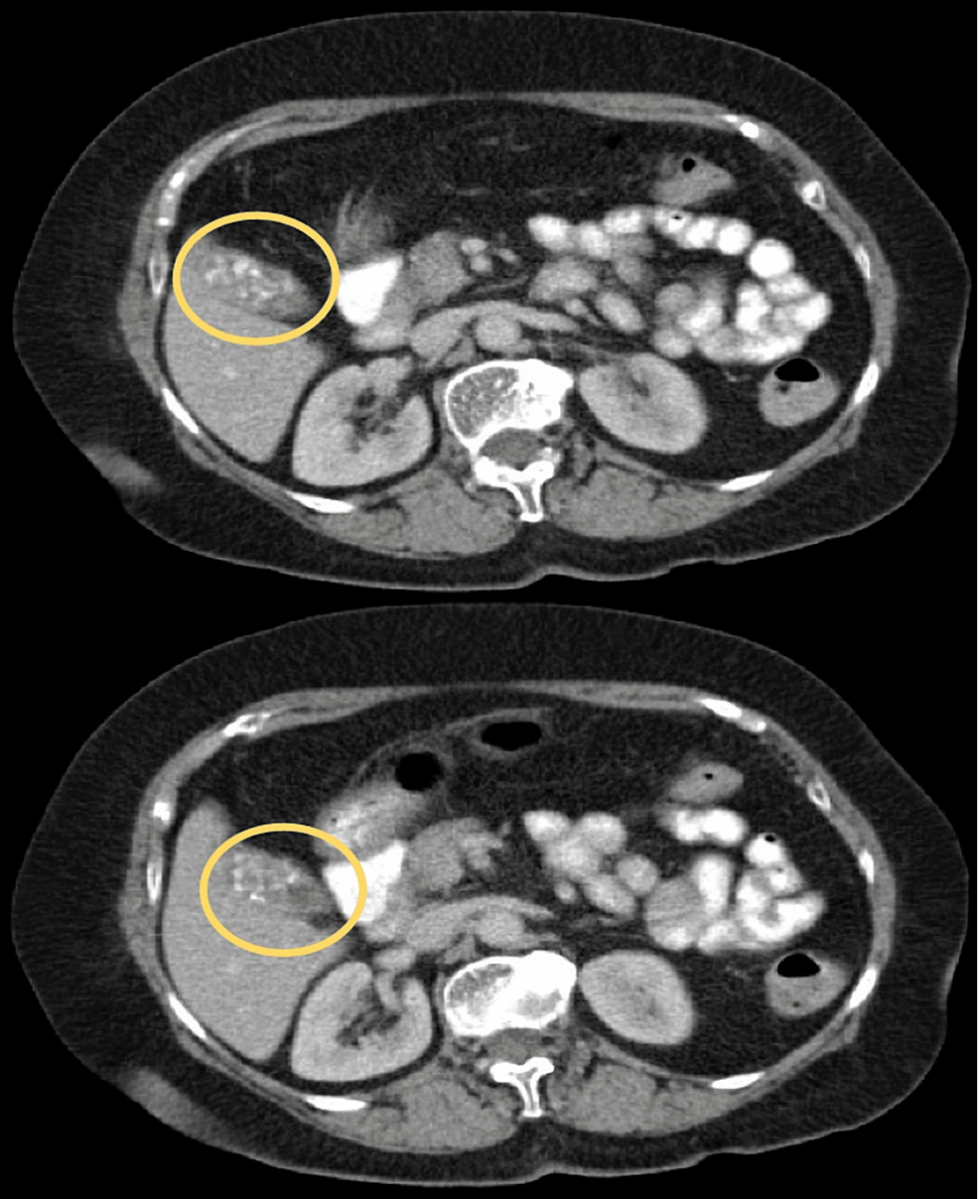
Pre-operative CT of the abdomen/pelvis demonstrating multiple impacted calcified gallstones. The yellow circles indicate multiple calcified gallstones that are impacted in the gallbladder.

The patient was referred to the surgical clinic after experiencing occasional abdominal discomfort. CT imaging revealed multiple gallstones, a stable 1.2-cm right inguinal lymph node, a 0.5-cm left inguinal node that decreased from 0.9 cm previously, and a 0.5-cm right external iliac node that decreased from 0.8 cm, without evidence of enlarging or new lymphadenopathy (Figure 1).

The patient then underwent elective cholecystectomy. She was noted intra-operatively to have chronic gallbladder inflammation but was otherwise uncomplicated. The post-operative course was unremarkable. The patient was observed and discharged on post-operative day 1.

**Figure 2:**
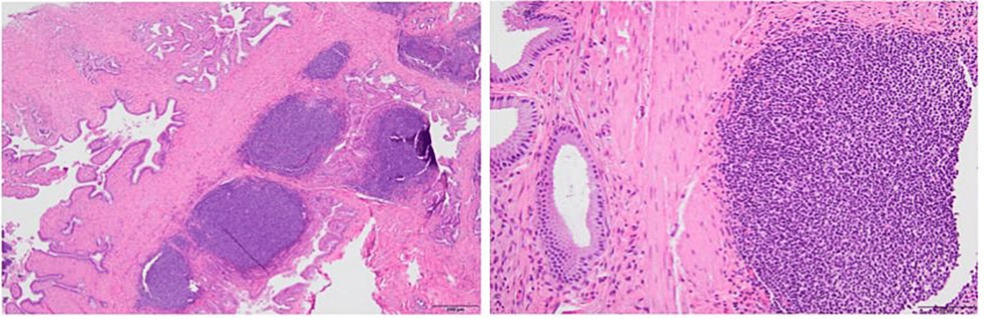
Histopathology of the gallbladder specimen after elective cholecystectomy revealing extranodal marginal zone mucosa-associated lymphoid tissue lymphoma.

The patient followed up with the surgical clinic one week post-operatively. Pathology was reviewed, which revealed a final histological diagnosis of extranodal marginal zone MALT lymphoma of the gallbladder. Immunohistochemistry revealed atypical lymphocytes that were positive for CD20 and BCL-2 while negative for CD3, CD5, BCL-6, CD43, and cyclin D1. Ki67 proliferation index was approximately 5%, which was diagnostic of a small B-cell lymphoma that is favored to represent extranodal marginal zone lymphoma. Pathology also revealed cholelithiasis and chronic cholecystitis with metaplastic changes (Figure 2).

The patient followed up two weeks post-operatively with a medical oncologist, and after extensive discussion, the decision was made to continue observation with close monitoring without systemic chemotherapy. If there was development of systemic symptoms or progressive and/or symptomatic lymph node enlargement, systemic chemotherapy will be reconsidered.

## Discussion

MALT lymphoma is a systemic disease that can manifest in multiple organs, but it is most commonly found in the gastrointestinal tract, salivary glands, thyroid gland, breast, and skin. However, MALT lymphoma of the gallbladder is exceedingly rare. The most common primary malignancy of the gallbladder is adenocarcinoma, while lymphoma accounts for only 0.1-0.2% of cases [3]. There appears to be a female predominance in these very few cases [4].

Prior to the diagnosis of primary MALT lymphoma of the gallbladder as a distinct disease process, histopathology findings of severe lymphocytic infiltration with the presence of lymphoid follicles in the gallbladder were previously diagnosed as chronic lymphocytic cholecystitis or lymphoid follicular cholecystitis [5]. Multiple theories exist regarding the pathophysiology of gallbladder lymphoma given the absence of lymphatic tissue. One theory suggests that chronic inflammation or bacterial infection results in transmural infiltration of lymphocytes and follicle formation. Another theory suggests that antigenic stimulation results in chromosomal translocation leading to synthesis of a fusion protein, API2-MALT1, an anti-apoptotic protein that can evade cell death and proliferate without requiring antigenic stimulation [3].

According to a study from 2015, CT or MRI findings suggesting lymphoma include homogenous submucosal thickening of the gallbladder wall with a preserved mucosal surface. Female predominance is apparent in patients diagnosed with extranodal marginal zone lymphoma of the gallbladder, similar to other anatomic sites [1]. There is currently no definitive method to diagnose gallbladder lymphoma pre-operatively. Therefore, all cases have been diagnosed post-operatively based on histopathological examination of gallbladder specimens [6].

Multiple studies have suggested that cholecystectomy alone is sufficient for long-term disease-free survival in these patients with gallbladder lymphoma. Extranodal marginal zone lymphoma of the gallbladder has a very good prognosis with early diagnosis and complete resection with cholecystectomy [4].

## Conclusions

Although extranodal marginal zone MALT lymphoma of the gallbladder represents a rare occurrence with clinical features similar to that of acute cholecystitis with abdominal pain, fever, nausea, and vomiting, it should be considered separately in the differential diagnosis. Multiple other cases suggest that extranodal marginal zone MALT lymphoma of the gallbladder will present as an incidental diagnosis on histopathology after a cholecystectomy. Long-term disease-free survival is optimized with cholecystectomy alone without systemic chemotherapy in addition to long-term clinical surveillance for recurrence.
